# Association between preoperative albumin and length of hospital stay in non-cardiac surgery patients with pulmonary hypertension: A secondary retrospective analysis

**DOI:** 10.1097/MD.0000000000038442

**Published:** 2024-06-07

**Authors:** Shu Wang, Zhouya Xue, Dan Su, Lin Ji, Yuanyuan Gao

**Affiliations:** a Affiliated Hospital 6 of Nantong University, Department of Anesthesiology, Yancheng City, Jiangsu Province, China; b Affiliated Yancheng Third People’s Hospital, Department of Anesthesiology, Yancheng City, Jiangsu Province, China; c Affiliated The First people's Hospital of Yancheng, Department of Anesthesiology, Yancheng City, Jiangsu Province, China; d Affiliated The Yancheng Clinical College of Xuzhou Medical University, Department of Anesthesiology, Yancheng City, Jiangsu Province, China.

**Keywords:** endothelial, length of hospital stay, preoperative serum albumin, pulmonary hypertension

## Abstract

To explore the risk factors affecting the length of hospital stay (LOS) as well as to examine the relationship between preoperative serum albumin levels and LOS following non-cardiac, non-obstetric surgery in patients with pulmonary hypertension (PHTN). This study represents a secondary retrospective analysis based on 287 non-cardiac, non-obstetric procedures performed on 195 PTHN patients at a single institution in the USA between 2007 and 2013. The primary outcome was the LOS. We conducted a multiple logistic regression analysis to compare the LOS between the 2 groups, divided at a serum albumin level of 3.5 g/dL. After adjusting for multiple covariates, the ORs for the long length of stay (LOS > 7 days) for the high group(albumin > 3.5 g/dL) compared with the low group (albumin ≤ 3.5 g/dL) were 0.35 (95%CI: 0.21~0.6), 0.41 (95%CI: 0.22 ~0.76), 0.41 (95%CI: 0.18~0.94) from model 2 to model 4. The stratified analysis results indicate that these findings are stable (*p* for trend > 0.05). In this study, it was observed that low levels of preoperative albumin were associated with an increased risk of prolonged hospital stay after non-cardiac, non-obstetric surgery in patients with PHTN. This implies that optimizing preoperative nutrition could potentially reduce the LOS for non-cardiac, non-obstetric surgery in patients with PHTN.

## 1. Introduction

Pulmonary hypertension (PTHN) is defined as a serious cardiovascular disease, characterized by a mean pulmonary artery pressure >20 mm Hg and resulting in increased resistance in the pulmonary circulation.^[1]^ Major Pulmonary Hypertension Clinical Registry studies have shown incidence rates of PHTN to range from 1.1 to 7.6 cases per million adults per year, with prevalence rates ranging from 11 to 26 cases per million adults.^[2]^ Limitations in functional status (FS) significantly impact patients’ quality of life, activities of daily living, and employment, thus placing a heavy burden on families and society.^[3]^ If left untreated, PTHN will progress to right heart failure and death.^[4]^ With advances in medicine, patients with PHTN are now more frequently undergoing non-cardiac, non-obstetric surgery. Patients with PHTN, particularly pulmonary arterial hypertension (PAH),^[5]^ who undergo surgery or anesthesia, face greater risks.^[6–8]^ The treatment and care of patients with PHTN during non-cardiac, non-obstetric surgery constitute a complex process, involving a multitude of factors.

Certain nutritional changes may trigger or worsen the progression of PHTN.^[9]^ Albumin, a 69 kDa protein, comprises more than half of the total serum protein composition. Synthesized by the liver, it is essential for maintaining blood volume, fluid regulation, nutrient transportation, and blood osmotic pressure regulation. It plays a vital role in the body, and variations in its levels can significantly influence patient recovery and length of stay. Hypoalbuminemia is recognized as a strong prognostic marker in the general population and in many pathologic settings, primarily due to malnutrition and inflammation.^[10]^ Previous studies have demonstrated that serum albumin concentration is an independent prognostic indicator in patients with PHTN.^[11]^

It is widely acknowledged that longer hospital stays are associated with higher economic costs, entailing more healthcare resources and services, thus leading to increased healthcare costs and lost work time and income for patients and families. Through preoperative nutritional optimization, certain conditions that would otherwise require a longer hospital stay can be addressed more swiftly, thus reducing the financial burden. Length of hospital stay (LOS) is often utilized as a proxy indicator of a patient health status during hospital treatment.^[12]^ A previous study revealed that heart failure patients with low albumin experienced longer hospital stays than those with high albumin.^[13]^ A retrospective cohort study in Thailand determined that gastrointestinal surgical patients with severe hypoalbuminemia faced a significantly higher risk of postoperative in-hospital death and longer hospital stays.^[14]^ In addition, preoperative serum albumin has been identified as independently associated with length of stay after radical cystectomy.^[15]^ However, the correlation between preoperative albumin levels and LOS after non-cardiac, non-obstetric surgery in people with PHTN remains unexplored.

The study of the relationship between preoperative serum albumin levels and LOS is important for improving the prognosis and reducing the LOS in patients with PHTN. In this study, we hypothesized a negative correlation between albumin levels and LOS after non-cardiac, non-obstetric surgery in patients with concomitant severe PHTN.

## 2. Methods

### 2.1. Data sources

This is a retrospective study, and the original data are accessible from the DRYAD Data Repository (DOI: 10.5061/dryad.9236ng5). The original study received approval from the University of Washington Institutional Review Board, which waived the informed consent requirement. Since Aalap C et al^[16]^ uploaded and transferred ownership of the raw data to the DRYAD website, we utilized the data for secondary analyses under various assumptions. This study adhered to the STROBE (Strengthening the Reporting of Observational Studies in Epidemiology) statement.

### 2.2. Study population

**Figure 1.**
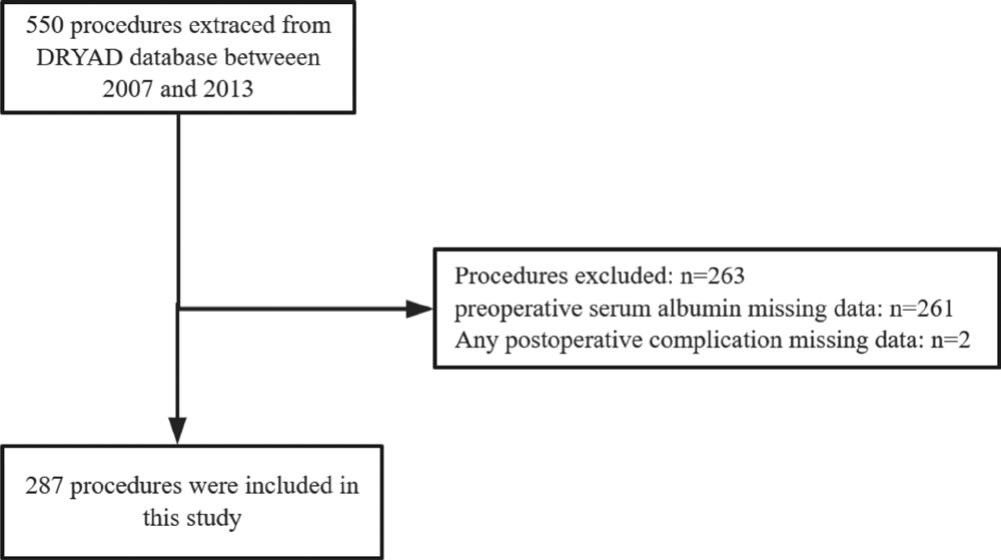
Flowchart of the present study.

We conducted a secondary analysis of 550 non-cardiac, non-obstetric procedures performed on 370 PHTN patients at an institution of the University of Washington between 2007 and 2013. All patients had a documented diagnosis of PHTN of any class or severity, as determined by echocardiography (ECHO) or cardiac catheterization (mean pulmonary artery pressure ≥ 25 mm Hg at rest and a pulmonary capillary wedge pressure ≤ 15 mm Hg), within 1 year prior to surgery. If multiple ECHO scans had been performed, the study closest to the date of surgery was selected for analysis. Methods of anesthesia included general anesthesia, local anesthesia, deep sedation, or monitored anesthesia care. Data were included only for the primary surgery in cases where patients underwent multiple surgeries during a single hospital stay. Specific inclusion criteria are detailed in the article written by Aalap C et al.^[16]^ Initially, 289 patients with preoperative serum albumin data were included. Subsequently, we excluded patients with missing data regarding any postoperative complication (n = 2). Ultimately, the study included 287 cases involving 195 patients. The detailed flow chart for patient recruitment is shown in Figure 1.

### 2.3. Variables

The exposure factor was the preoperative serum albumin level, and the main outcome measured was the length of stay (LOS, defined as the time interval from date of surgery to date of discharge or death in-hospital). Hospital stays exceeding 7 days were considered “long length of hospital stay.”^[17]^ Covariates included clinical data obtained from patients’ medical records. Demographic characteristics included sex, age, BMI. Surgical data included the number of procedures, ASA (American Society of Anesthesiologists) classification, anesthesia approach, procedure length, self-reported FS, open surgical approach, and the severity of PHTN.

Preoperative comorbidities recorded were smoking history, systemic hypertension, coronary artery disease (CAD), congestive heart failure (CHF), deep venous thrombosis(DVT), arrhythmia, asthma, chronic obstructive pulmonary disease (COPD), obstructive sleep apnea (OSA), diabetes mellitus (DM), renal failure, angina. Laboratory data also included creatinine, hemoglobin (Hgb), white blood cell (WBC), ejection fraction (EF). During the pre-anesthesia outpatient visit, all patients were requested to estimate the number of blocks they could walk and the number of stairs they could climb without experiencing any symptoms. This information was employed to categorize patients based on their MET (a measure of FS). Patients incapable of walking 4 blocks or climbing 2 flights of stairs were classified as having poor exercise tolerance (FS < 4 METs).^[5,18,19]^ Pulmonary systolic blood pressure of ≥ 59 mm Hg is classified as severe PHTN.^[20]^

### 2.4. Statistical analysis

Continuous variables were represented as means with standard deviations (SD) or medians with interquartile ranges (IQR), and categorical variables as numbers and percentages. Baseline characteristics were classified based on preoperative serum albumin level (3.5 g/L). Multivariate logistic regression was used to determine the odds ratios (ORs) and 95% confidence intervals (CIs) for the relationship between preoperative serum albumin level and the risk of long LOS (>7 days). An extended logistic regression model approach was used for different covariates-adjusted models. These potential confounders were chosen on the basis of previous scientific literature, or those exhibited significance in univariate analyses (*P* < .05). Four models were employed: model I as a crude model: univariable; model II modified with age and gender; model III, adjusted for model II plus ASA classification, surgical approach, self-reported FS, procedure length, severity of PHTN; and model IV, adjusted for model III plus BMI, number of procedures, anesthesia approach, current tobacco use, systemic hypertension, CAD, CHF, DVT, arrhythmia, asthma, COPD, diabetes, renal failure, angina, EF, creatinine, WBC, Hgb. Subgroup analyses were performed using stratified logistic regression models. Interaction among subgroups was examined using the likelihood ratio test. All analyses were performed using the statistical software packages R (http://www.R-project.org, The R Foundation) and Free Statistics software version 1.7.1. A *P* value of <.05 was considered statistically significant.

## 3. Results

### 3.1. Study population

In the original study, 550 non-cardiac, non-obstetric procedures performed on 370 PHTN patients at an institution of the University of Washington between 2007 and 2013 were included. We further excluded cases with missing albumin data (n = 261) and cases with any postoperative comorbidity (n = 2). A total of 287 procedures performed on 195 patients were ultimately included. The detailed flow chart is depicted in Figure 1.

### 3.2. Baseline characteristics of study subjects

A total of 287 procedures were included in the study. Of these, 151 had an albumin over 3.5 g/dL and 125 (43.6%) were female. The median LOS was 3.9 days (range, 1.0–12.2 days) in the group with an albumin level below 3.5 g/dL, while the median LOS was 1.4 days (range, 0.4–6.2 days) in the group with an albumin level above 3.5 g/dL. The mean age was 58.7 years old (SD13.8). The cases in the group with albumin below 3.5 g/dL tend to be more with poor self-reported FS. Some differences existed between the albumin groups in terms of various covariates(poor self-reported FS, renal failure, Hgb). Detailed baseline characteristics are shown in Table 1.

**Table 1:** Baseline characteristics of participants from DRYAD database by categories of preoperative albumin levels.

Characteristic	Total	Albumin, g/L		*P*
		Q1(≤3.5)	Q2(>3.5)	
Participants(*n*)	287	136	151	
LOS, Median (IQR)	2.3 (0.5, 9.0)	3.9 (1.0, 12.2)	1.4 (0.4, 6.2)	<.001
Gender, *n* (%)				.674
Male	162 (56.4)	75 (55.1)	87 (57.6)	
Female	125 (43.6)	61 (44.9)	64 (42.4)	
Age (yr), Mean ± SD	58.7 ± 13.8	59.2 ± 13.1	58.3 ± 14.5	.553
BMI (kg/m^2^), Mean ± SD	31.5 ± 10.9	31.5 ± 12.2	31.4 ± 9.6	.984
Number of procedures, *n* (%)				.273
1	194 (67.6)	86 (63.2)	108 (71.5)	
2	51 (17.8)	26 (19.1)	25 (16.6)	
3+	42 (14.6)	24 (17.6)	18 (11.9)	
ASA classification, n (%)				.288
I–III	213 (74.2)	97 (71.3)	116 (76.8)	
IV	74 (25.8)	39 (28.7)	35 (23.2)	
Anesthesia approach, *n* (%)				.26
General anesthesia	228 (80.0)	111 (82.8)	117 (77.5)	
Regional anesthesia, deep sedation, or monitored anesthesia care	57 (20.0)	23 (17.2)	34 (22.5)	
Procedure length (min), Median (IQR)	79.0 (34.0, 155.0)	79.0 (42.0, 156.0)	81.5 (29.0, 140.0)	.366
Self-reported FS, *n* (%)				.001
Normal	139 (48.4)	52 (38.2)	87 (57.6)	
Poor	148 (51.6)	84 (61.8)	64 (42.4)	
Surgical approach, *n* (%)				.703
Open surgery	130 (45.3)	60 (44.1)	70 (46.4)	
Non-open surgery	157 (54.7)	76 (55.9)	81 (53.6)	
Severity of PHTN, *n* (%)				.515
Non-severe	255 (92.7)	121 (91.7)	134 (93.7)	
Severe	20 (7.3)	11 (8.3)	9 (6.3)	
Current tobacco use, *n* (%)				.965
No	264 (92.0)	125 (91.9)	139 (92.1)	
Yes	23 (8.0)	11 (8.1)	12 (7.9)	
Systemic hypertension, *n* (%)				.986
No	112 (39.0)	53 (39)	59 (39.1)	
Yes	175 (61.0)	83 (61)	92 (60.9)	
CAD, *n* (%)				.774
No	196 (69.3)	91 (68.4)	105 (70)	
Yes	87 (30.7)	42 (31.6)	45 (30)	
CHF, *n* (%)				.417
No	198 (69.0)	97 (71.3)	101 (66.9)	
Yes	89 (31.0)	39 (28.7)	50 (33.1)	
DVT, *n* (%)				.978
No	270 (94.1)	128 (94.1)	142 (94)	
Yes	17 (5.9)	8 (5.9)	9 (6)	
Arrhythmia, *n* (%)				.96
No	165 (57.5)	78 (57.4)	87 (57.6)	
Yes	122 (42.5)	58 (42.6)	64 (42.4)	
Asthma, *n* (%)				.838
No	247 (86.4)	116 (85.9)	131 (86.8)	
Yes	39 (13.6)	19 (14.1)	20 (13.2)	
COPD, *n* (%)				.762
No	242 (84.6)	116 (85.3)	126 (84)	
Yes	44 (15.4)	20 (14.7)	24 (16)	
OSA, *n* (%)				.345
No	223 (77.7)	109 (80.1)	114 (75.5)	
Yes	64 (22.3)	27 (19.9)	37 (24.5)	
Diabetes, *n* (%)				.151
No	211 (73.8)	95 (69.9)	116 (77.3)	
Yes	75 (26.2)	41 (30.1)	34 (22.7)	
Renal failure, *n* (%)				.012
No	204 (71.1)	87 (64)	117 (77.5)	
Yes	83 (28.9)	49 (36)	34 (22.5)	
Angina, *n* (%)				.85
No	265 (92.3)	126 (92.6)	139 (92.1)	
Yes	22 (7.7)	10 (7.4)	12 (7.9)	
Creatinine (µmol/L), Median (IQR)	1.1 (0.8, 1.6)	1.2 (0.9, 1.9)	1.0 (0.8, 1.5)	.079
Hgb (g/L), Mean ± SD	11.7 ± 2.7	10.8 ± 2.6	12.5 ± 2.5	<.001
WBC (10^9/L), Median (IQR)	7.3 (5.6, 9.5)	7.4 (5.6, 9.5)	7.3 (5.6, 9.5)	.759
EF value (%), Mean ± SD	57.1 ± 15.8	57.9 ± 15.9	56.3 ± 15.7	.404
Any complication within 30 d, n (%)				.262
No	193 (67.2)	87 (64)	106 (70.2)	
Yes	94 (32.8)	49 (36)	45 (29.8)	

Data are shown as mean ± SD, median (IQR), or n (%).

ASA = American Society of Anesthesiologists, BMI = body mass index, CAD = coronary artery disease, CHF = congestive heart failure, COPD = chronic obstructive pulmonary disease, DVT = deep venous thromboembolism, EF = ejection fraction, FS = functional status, LOS = length of hospital stay, OSA = obstructive sleep apnea.

### 3.3. Association of covariates and risk of long LOS (>7 days)

Univariate analysis was employed to identify the risk factors for LOS > 7 days in non-cardiac, non-obstetric surgery patients with PHTN. ASA classification, procedure length, poor self-reported FS, open surgical approach, severe PHTN, Hgb were found to be significantly associated with the risk of a LOS > 7 days (*P* < .05). Other factors did not show a significant association with the risk of a LOS > 7 days (Table 2).

**Table 2:** Univariate analysis of risk factor associated with long length of stay (LOS > 7 d).

Variable	OR (95%CI)	*P* value
Gender		
Male	Reference	
Female	0.94 (0.57~1.57)	.817
Age	1 (0.98~1.02)	.982
BMI (kg/m^2^)	1.01 (0.98~1.03)	.568
Number of procedures		
1	Reference	
2	0.85 (0.43~1.68)	.631
3+	1 (0.49~2.06)	.998
ASA classification	2.57 (1.48~4.46)	.001
Anesthesia approach		
General anesthesia	Reference	
Regional anesthesia, deep sedation, or monitored anesthesia care	1.46 (0.79~2.69)	.222
Procedure length (min)	1.01 (1~1.01)	<.001
Self-reported FS		
Normal	Reference	
Poor	2.63 (1.55~4.46)	<.001
Surgical approach		
Open surgery	Reference	
Non-open surgery	3.76 (2.14~6.62)	<.001
Severity of PHTN		
Non-severe	Reference	
Severe	2.99 (1.19~7.51)	.02
Current tobacco use		
No	Reference	
Yes	1.01 (0.4~2.54)	.989
Systemic hypertension		
No	Reference	
Yes	0.62 (0.37~1.03)	.064
CAD		
No	Reference	
Yes	1.46 (0.85~2.5)	.172
CHF		
No	Reference	
Yes	0.79 (0.45~1.38)	.409
DVT		
No	Reference	
Yes	2.15 (0.8~5.77)	.129
Arrhythmia		
No	Reference	
Yes	1 (0.6~1.67)	.996
Asthma		
No	Reference	
Yes	1.19 (0.58~2.45)	.633
COPD		
No	Reference	
Yes	1.4 (0.72~2.76)	.324
OSA		
No	Reference	
Yes	1.06 (0.58~1.93)	.853
Diabetes		
No	Reference	
Yes	0.71 (0.39~1.29)	.266
Renal failure		
No	Reference	
Yes	1.07 (0.62~1.86)	.812
Angina		
No	Reference	
Yes	1.08 (0.42~2.75)	.873
Creatinine (µmol/L)	1.05 (0.92~1.2)	.481
Hgb (g/L)	0.77 (0.68~0.87)	<.001
WBC (10^9/L)	1 (0.98~1.02)	.658
EF value (%)	0.99 (0.98~1)	.054

ASA = American Society of Anesthesiologists, BMI = body mass index, CAD = coronary artery disease, CHF = congestive heart failure, COPD = chronic obstructive pulmonary disease, DVT = deep venous thromboembolism, EF = ejection fraction, FS = functional status, OSA = obstructive sleep apnea.

### 3.4. Association between preoperative serum albumin and a long LOS (>7 days)

Table 3 demonstrates the association between preoperative albumin level and risk of LOS > 7 days across multiple models. In the unadjusted model, the odds ratio (OR) for the high group (albumin > 3.5 g/dL) relative to the low group (albumin ≤ 3.5 g/dL) was 0.34 (95% CI 0.2–0.58). After adjusting for variables, the respective ORs were 0.35 (95% CI: 0.21–0.6), 0.41 (95% CI: 0.22–0.76), and 0.41 (95% CI: 0.18–0.94) in models II to IV.

**Table 3:** Association between preoperative albumin levels and long length of hospital stay (LOS > 7 d) in the database from DRYAD.

Variable	n.total	n.event_%	Model I	*P*	Model II	*P*	Model III	*P*	Model IV	*P*
			OR(95%CI)		OR(95%CI)		OR(95%CI)		OR(95%CI)	
Albumin(g/L)	287	87 (30.3)	0.34 (0.23~0.51)	<.001	0.34 (0.23~0.51)	<.001	0.37 (0.23~0.6)	<.001	0.36 (0.19~0.69)	.002
Albumin(g/L)										
≤3.5	136	57 (41.9)	1 (Ref)		1 (Ref)		1 (Ref)		1 (Ref)	
>3.5	151	30 (19.9)	0.34 (0.2~0.58)	<.001	0.35 (0.21~0.6)	<.001	0.41 (0.22~0.76)	.005	0.41 (0.18~0.94)	.035

Model I not adjusted.

Model II adjusts for age and gender.

Model III adjusts for Model II + ASA classification, surgical approach, self-reported FS, procedure length, severity of PHTN.

Model IV adjusts for Model III + BMI, number of procedures, anesthesia approach, current tobacco use, systemic hypertension, CAD, CHF, DVT, arrhythmia, asthma, COPD, diabetes, renal failure, angina, EF, creatinine, WBC, Hgb.

### 3.5. Stratified analyze based on additional variables

**Figure 2.**
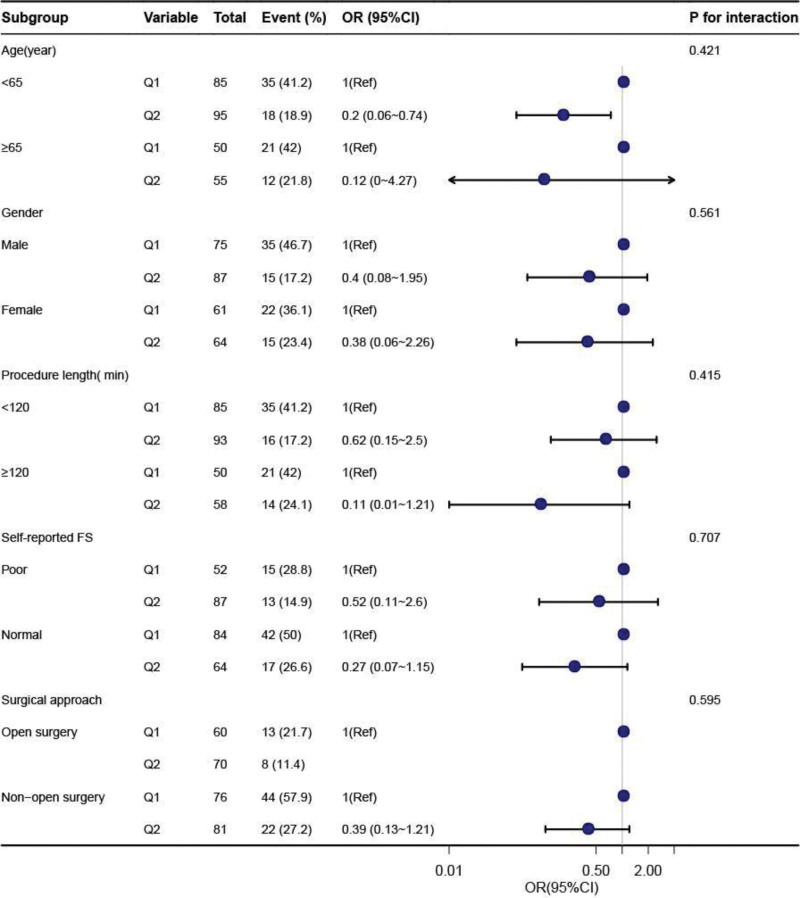
Subgroup analysis of the association between preoperative albumin levels and long length of hospital stay (LOS > 7 d) in the data from DRYAD. Adjusted for age, gender, ASA classification, surgical approach, self-reported FS, procedure length, severity of PHTN, BMI, number of procedures, anesthesia.approach, current tobacco use, systemic hypertension, CAD, CHF, VT, arrhythmia, asthma, COPD, diabetes, renal failure, angina, EF, creatinine, WBC, Hgb. ASA = American Society of Anesthesiologists, BMI = body mass index, CAD = coronary artery disease, CHF = congestive heart failure, CI = confidence interval, COPD = chronic obstructive pulmonary disease, FS = functional status, OR = odds ratio, PHTN = pulmonary hypertension.

The stratified analyses were conducted to examine whether the association between albumin level and the risk of a LOS > 7days was stable in different subgroups. After stratification by age, gender, procedure length, self-reported FS, and open surgical approach, no significant interactions were observed in any of the groups (Fig. 2).

## 4. Discussion

In this retrospective study, we found that preoperative serum albumin levels were negatively associated with the LOS after non-cardiac, non-obstetric surgery in patients with PHTN, and the results remained stable after adjusting for several covariates.

The physiological properties of serum albumin encompass anti-inflammatory, antioxidant, anticoagulant, and antiplatelet aggregation activities, as well as colloid osmotic effects. Albumin is synthesized by the liver and is secreted into the plasma, as well as into and out of the blood vessels.^[21]^ However, its serum level is not solely determined by liver function. Nutritional status, hepatic FS, and inflammatory status are among the factors that can influence serum albumin levels.^[22]^ Hypoalbuminemia has been identified as a prognostic indicator for various critical cardiovascular diseases, including acute heart failure,^[23,24]^ congenital heart disease,^[25]^ acute coronary syndrome,^[26]^ and PAH.^[11,27]^ As a prognostic tool for assessing nutritional status and surgical outcomes, preoperative serum albumin serves as an easily accessible and affordable marker to identify patients at increased risk for prolonged LOS.^[15]^ Several studies have demonstrated a similar correlation between lower preoperative serum albumin levels and prolonged LOS in various surgical procedures.^[28–30]^ Low albumin levels could reflect abnormal protein metabolism or malnutrition, potentially leading to reduced immune function, decreased tissue repair, among other issues, thereby prolonging the length of hospitalization.

In patients with PAH, low albumin levels could be associated with the development and progression of the condition. Increased right atrial pressure can lead to an increased transcapillary escape of albumin.^[31]^ Animal studies have shown that lower levels of albumin increase the permeability of the pulmonary vascular system, thereby exacerbating pulmonary edema.^[32]^ Capillary dysfunction represents a significant mechanism in the development of PHTN, with endothelial dysfunction and capillary leakage being key factors in albumin catabolism.^[22]^ Studies have demonstrated that endothelial cells (ECs) play a crucial role in the onset and progression of PAH.^[33]^ The healthy endothelial monolayer lining the inner wall of blood vessels is responsible for regulating the flow of fluids, proteins, and blood cells across the vessel wall into parenchymal tissues, maintaining vascular tone and integrity, and exerting antithrombotic and anti-inflammatory effects on the vascular bed.^[34]^ The stability of endothelial function in the vasculature is critical to the normal operation of circulatory function.^[35]^ The current understanding of vascular permeability largely relies on the Starling principle. According to this principle, fluid transport to and from the interstitial spaces of peripheral tissues is governed by an equilibrium between opposing pressures and hydrostatic pressure. This balance is particularly influenced by the nature of the vascular barrier.^[36]^ Recent studies indicate that the endothelial glycocalyx, forming part of the vascular barrier alongside ECs (located on the luminal side of the healthy vascular system and at least 200 nm thick), is vital for vascular permeability. While water and electrolytes can pass freely through the glycocalyx, plasma proteins, especially albumin, have a strong interaction with it.^[36]^ Plasma components bind to and become embedded within the structural elements of the glycocalyx, forming what is known as the endothelial surface layer. This layer serves as the actual interface between the blood flowing in the body and the EC membrane. Albumin contributes to the protection of ECs by enhancing interactions with the endothelial glycocalyx.^[37]^

Under normal conditions, the endothelium facilitates vasodilation and prevents thrombosis and leukocyte adhesion. ECs synthesize a variety of substances, including prostacyclin, NO, endothelin, TM, and heparan sulfate, to regulate pulmonary blood flow and vascular resistance.^[38]^ However, in patients with PAH, ECs tend to be damaged and/or dysfunctional.^[39–41]^ Injury to ECs can increase vascular permeability, leading to albumin leakage from blood vessels into surrounding tissues and a consequent decline in plasma albumin levels. Therefore, any impairment in EC function could reduce the protective impact of albumin. In conclusion, there is a close relationship between albumin levels and EC injury. EC injury can result in decreased albumin levels, impair albumin-EC interactions, and reduce the protective effects of albumin. Low protein levels may lead to exacerbation by exacerbating the pathophysiological process of PHTN, thereby prolonging hospitalization. In other words, hypoproteinemia may act as a mediator in the relationship between PHTN and length of hospitalization.

Our study has several limitations that cannot be ignored. First, as this is a retrospective study, it can only demonstrate a correlation between serum albumin levels and LOS, rather than establish causation. Secondly, since the data were obtained from a single center, they are limited in size and may not be representative of the broader population with PHTN. Thirdly, this is a retrospective observational study and there may be incomplete, inaccurate or missing data recording, so the results may be biased. Finally, this study applies to the U.S. population with PHTN, and it is not certain that it is appropriate for other regions, and further research is needed.

## 5. Conclusions

The findings of this study suggest that preoperative serum albumin levels and the LOS in patients with PHTN undergoing non-cardiac, non-obstetric surgery are related. In our clinical work, we may be able to increase albumin levels through nutritional support, thereby improving outcomes and reducing the LOS. In addition, we will conduct further multicenter studies to further explore the relationship between preoperative albumin levels and the LOS in the PHTN population.

## Acknowledgments

We thank all the participants for their excellent contributions to guarantee the completion of the study. We thank Free Statistics team for providing technical assistance and valuable tools for data analysis and visualization.

## Author contributions

**Conceptualization:** Shu Wang, Zhouya Xue, Lin Ji, Yuanyuan Gao.

**Data curation:** Dan Su.

**Formal analysis:** Shu Wang, Zhouya Xue, Dan Su.

**Investigation:** Shu Wang, Zhouya Xue.

**Methodology:** Shu Wang, Zhouya Xue, Dan Su.

**Supervision:** Lin Ji, Yuanyuan Gao.

**Writing – original draft:** Shu Wang, Zhouya Xue.

**Writing – review & editing:** Lin Ji, Yuanyuan Gao.
